# Predictors of public attitudes in Saudi Arabia toward people who stutter

**DOI:** 10.1371/journal.pone.0295029

**Published:** 2023-12-21

**Authors:** Nisreen Naser Al Awaji, Reem Fouzan Alfouzan, Afnan Razen Almutairi, Eman M. Mortada

**Affiliations:** 1 Department of Health Communication Sciences, College of Health and Rehabilitation Sciences, Princess Nourah bint Abdulrahman University, Riyadh, Saudi Arabia; 2 Formerly of Department of Health Communication Sciences, College of Health and Rehabilitation Sciences, Princess Nourah bint Abdulrahman University, Riyadh, Saudi Arabia; 3 Department of Health Sciences, College of Health and Rehabilitation Sciences, Princess Nourah bint Abdulrahman University, Riyadh, Saudi Arabia; University of Sharjah, UNITED ARAB EMIRATES

## Abstract

**Purpose:**

The Public Opinion Survey of Human Attributes-Stuttering (POSHA-S) was used to measure the attitudes of the general population in Saudi Arabia toward people who stutter (PWS) and to identify the predictors of the overall stuttering score (OSS).

**Method:**

A total of 404 adults from Saudi Arabia (16.8% male and 83.2% female) completed an online POSHA-S questionnaire.

**Results:**

The attitudes of adults in Saudi Arabia were similar to those of other samples worldwide. Working status, income, and multilingualism were substantial predictors of the OSS.

**Conclusion:**

Saudi adults have positive impressions, beliefs, and self-reactions to PWS. However, their knowledge of stuttering tends to be limited. Therefore, campaigns conducted to raise awareness of stuttering should adopt the most widely used sources of knowledge in the Saudi Arabian community (i.e., the Internet and social media). Sociodemographic variables predictive of positive versus negative OSS include working status and multilingualism. Unpredictive variables, that do not predict positive versus negative OSS, include age, gender, education, parental status, health, abilities, and income.

## Introduction

Stuttering is a multifactorial speech fluency disorder characterized by difficulty producing continuous, smooth, and effortless speech. It can be classified into developmental and acquired stuttering. Studies have reported that the prevalence of developmental stuttering is 5% of the general population, with a 1% incidence over the lifespan [1]. In other words, of all children who stutter (or have developmental stuttering), 80% usually recover during childhood, leaving 20% who do not recover during childhood and experience persistent developmental stuttering [1, 2] Many other rare cases of acquired stuttering are not included in this group and occur as a secondary result of emotional trauma or brain injury.

Numerous studies have reported negative attitudes among nonstuttering adults toward people who stutter (PWS) in samples around the world [3–7]. Perceptions of PWS, although varied, are stereotypical in regarding them as quiet, fearful, worthless, nervous, shy, withdrawn, restrained, or introverted. Such negative attitudes place PWS in stigmatized or compromised categories, making them feel discredited and unworthy, limiting their abilities, choices, and opportunities [8] Stigmatized attitudes can also lead to academic underachievement, compromised quality of life, negative self-esteem, unemployment or underemployment, and adverse social reactions, such as rejection, bullying, taunting, and social injury [6, 9, 10] Concerning career possibilities, people who do not stutter are more likely than PWS to work in positions requiring communication skills [11]. Furthermore, PWS are seen as less intelligent than their non-stuttering co-workers [11]. Several studies have shown that society’s understanding of communication disorders, including stuttering, significantly influences attitudes toward PWS [12, 13] Gender, age, education, and culture also affect non-stutterers’ attitudes toward PWS.

Various studies have reported cultural differences in attitudes toward PWS. For example, Western cultures have shown more positive attitudes than other cultures, such as those in Asia [14–16], Africa [17], and the Middle East [3, 4]. Üstün-Yavuz [18] recently demonstrated that the overall stuttering score (OSS), which is a measure of general attitudes toward stuttering based on the Public Opinion Survey of Human Attributes-Stuttering (POSHA-S), was the highest for British participants (mean = 30), followed by Arab participants (mean = 21) and Chinese participants (mean = 13). However, studies comparing the attitudes of adults and children found that the attitudes of children varied less across diverse cultures than those of adults [19, 20].

Examining instructors’ views on stuttering across two distinct ethnicities (Americans and Arabs), Irani [21] observed that American teachers expressed more favorable attitudes than Arab teachers, who characterized PWS as unsociable, angry, mentally unstable, untrustworthy, and lacking a sense of humor. In their studies conducted in Egypt, Arafa et al. [3] and Nabieh El-Adawy [4] reported fewer positive attitudes from Egyptians toward PWS than other populations worldwide. Another study conducted in Kuwait reported that despite limited social knowledge and a lack of full acceptance of the disorder, the Kuwaiti community generally had positive attitudes toward stuttering and PWS [22].

To the best of our knowledge, no studies have been conducted in the Kingdom of Saudi Arabia (KSA) to determine the general population’s attitudes toward PWS. The current study was carried out to identify OSS predictors and evaluate the attitudes, beliefs, and knowledge of Saudi people regarding PWS using the POSHA-S questionnaire.

### Saudi Arabian context

This section offers a brief glance at the social, economic, and political context in KSA. The KSA, located on the Arabian Peninsula in western Asia, is the second-largest country in the Arabic world and the fifth-most populous country in Asia. The KSA is a significant regional and middle power, with the 18^th^ largest economy in the world and the largest in the Middle East. A substantial percentage (67%) of the KSA’s 34.8 million inhabitants is aged 15–34 years, making it one of the youngest populations in the world. Among this young population, 98.4% of females and 97.6% of males participate in social networks, professional networks, or the use of social media [23].

In 2017, Saudi Arabia announced a series of broad socioeconomic reforms, known as Vision 2030, to reduce the country’s dependence on oil, increase economic growth, and improve several public services sectors, such as education, healthcare, infrastructure, entertainment, and tourism [24]. Through Vision 2030, the community is encouraged to develop civic values, such as respect, community service, responsibility, social justice, creativity, moderation, tolerance, and skills required for self-development and success in future careers. Many public services and social changes are underway to realize Vision 2030, including programs to socially empower Saudi women.

## Materials and method

### Public attitude measure

The respondents’ attitudes toward stuttering were collected using the POSHA*-*S [25] and translated into Arabic [26]. The POSHA-S is a self-administered questionnaire developed as part of the International Project on Attitudes toward Human Attributes (IPATHA) [25] and has been widely used internationally.

The POSHA-S comprises three main sections:

1. The first section includes sociodemographic questions related to age, sex, years of education, social status, occupation, income, religion, native language, other known languages, and self-ratings of physical health, speaking, and learning ability.
2. The second section includes a general comparison of stuttering with four other anchor attributes (left-handedness, mental illness, obesity, and intelligence) using a 5-point Likert scale.
3. The third section evaluates respondents’ *beliefs* about and *self-reactions* to PWS and stuttering using a 3-point scale (no = 1, not sure = 2, and yes = 3). The *beliefs* subscores measure the following four components: traits or personality, potentials, causes, and sources of help. The *self-reaction* subscores measure the following four components: accommodating or helping, social distance or sympathy, knowledge and experience, and knowledge source.

**Fig 1:**
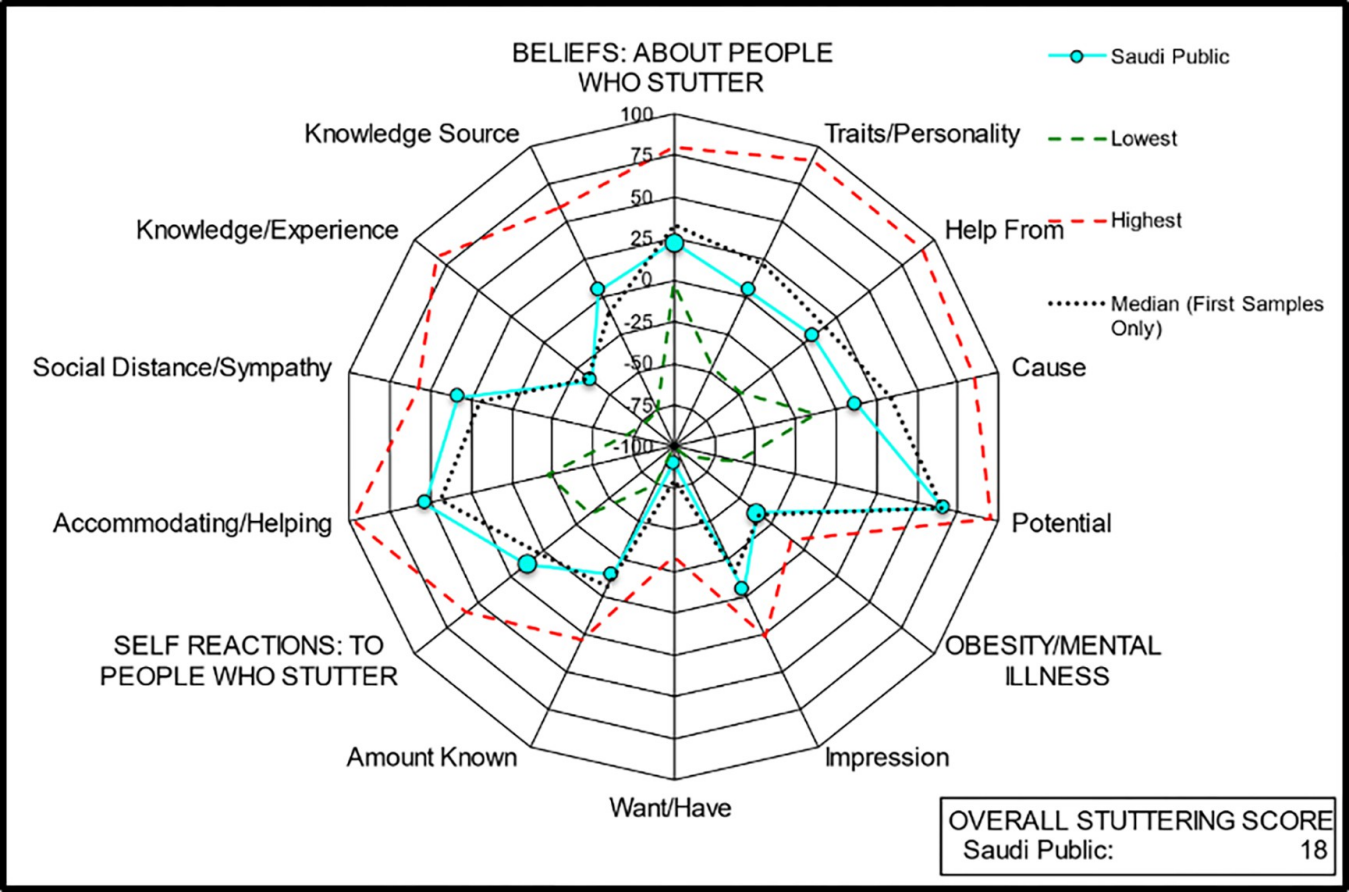
Summary of POSHA-S components, subscores, and OSS of participants in Saudi Arabia.

The means of the general and stuttering items were combined to generate components, and those of the components were combined to generate subscores, as shown below in Table 2 and Fig 1. The mean of the two subscores related to stuttering—beliefs and self-reactions—yielded the OSS. A third subscore combined three items regarding two other negatively perceived human attributes to generate an obesity/mental illness subscore, which compared stuttering with these attributes. The scores obtained from the abovementioned items were converted to a scale from −100 to +100, where 0 indicates a neutral rating. However, the mean scores for some items were reversed, such that the higher converted ratings indicated a more positive or better attitude (i.e., less agreement), whereas the lower scores indicated a less positive or worse attitude (i.e., more agreement). An example of a reversed item is “PWS are shy or fearful.” For such items, a high score indicates that the respondents do not agree that PWS are shy or fearful, which reflects a more positive, sensitive, or accurate attitude.

### Participants and sampling procedures

This population-based study used a cross-sectional research design and targeted adults in the KSA aged 18 years and older. After all necessary ethical approvals were obtained from the Internal Review Board (Log Number: 20–0512), the POSHA-S questionnaire was distributed through social media channels: Twitter, Facebook, WhatsApp, and Telegram. Obtained responses comprised of 404 adults (16.8% male and 83.2% female). A consent statement and question were added at the start of the survey, and before accessing the survey, respondents had to check a box indicating their consent and agreement to participate in the study. Additionally, respondents received guarantees about the privacy of their data and their freedom to leave the study at any time.

### Data analysis

A widely applied method was used for the data analysis [26–28] with the questionnaire data entered into and analyzed with Microsoft Excel. The converted mean ratings (ranging from −100 to +100) for each item, component, subscore, and OSS were reported.

To compare the KSA data to the POSHA-S database, percentiles relative to 268 sample means from the POSHA-S database were generated for the means of the Saudi sample and divided into the following quartiles (i.e., percentiles): the percentage of all Saudi mean ratings in the first or lowest quartile (0–25^th^ percentile), the interquartile range (25^th^–75^th^ percentile), and the fourth quartile range (75^th^–100^th^ percentile). The results were also displayed in a radial graph, where the mean ratings of the Saudi sample were compared with those of the 268 samples of the POSHA-S database, which comprised 19,702 respondents from 47 countries responding in 27 languages (circa March 2021). Thus, the highest (most positive or best) mean attitudes, lowest (least positive or worst) mean attitudes, and 50^th^ percentile observed to date in the POSHA-S sample are displayed in the graph and compared with the results of the Saudi sample.

Hierarchical multiple regression was used to explore the predictive role of all independent study domains on the OSS. Demographic variables were entered into the first block of the regression analysis, creating a model that included age (y), gender, education (y), marital status, parental status, working status, and income. Health/abilities were entered into the second block, and multilingualism into the third block. The fourth and fifth blocks included self-identification and no-person-known domains, respectively. In the sixth and seventh models, all subscales of the OSS—namely, beliefs about and self-reactions to PWS—were included in the respective model. All statistical tests were two-sided, and a *p*-value of ≤ .05 was considered statistically significant.

## Results

### Demographics of the respondents

Table 1 presents the demographic information of the 404 Saudi respondents who participated in this study. The table shows that the results were compared with the median values of the 268 samples analyzed in the POSHA-S database. Percentiles for the Saudi means generated from the 268 database means were also added to the table.

**Table 1:** Characteristics of the Saudi respondents: POSHA-S mean values of the Saudi respondents, mean values from the POSHA-S database samples (268 samples circa March 2021), and percentiles for the Saudi means relative to the POSHA-S database.

Variable	Characteristics	Saudi Respondents	Public POSHA-S samples (Median)	Public POSHA-S samples (Mean)	Saudi Sample Percentile
**Sample**		404	60	114	98
**Age**		26.97	36.88	36.54	36
**Gender (%)**	Male	16.83	37.50	38.00	27
	Female	83.17	62.50	62.00	73
**Years of Education**		14.29	14.48	14.05	40
**Marital Status (%)**	Married	36.63	54.72	52.40	45
	Single	63.37	45.28	47.60	55
**Parental Status (%)**	Parent	29.72	49.36	49.91	43
	Non-parent	70.28	50.64	50.09	57
**Working Status (%)**	Student	51.98	13.74	24.54	63
	Working	27.97	63.43	60.12	26
	Not working	18.56	6.10	12.81	89
	Retired	1.49	2.70	5.81	68
**Income**	Score	9	0	1	67
	Family/Friend	3.18	8	8	34
	Country-mean	3.18	0	0	53
**Health and Abilities**	Physical health	32	42	41	15
	Mental health	62	55	54	75
	Ability to learn.	70	56	54	82
	Ability to speak	66	62	58	66
**Multilingual**		94.31	51.68	45.87	84

The respondents had a mean age of 27.0 years and an average of 14.3 years of education, which is close to the POSHA-S database average (14.5 years). There was a high variation between the male and female percentages in the Saudi sample: approximately 83% of the participants were female, while approximately 17% were male. In addition, only 37% of the respondents were married, and 30% of these were parents. In comparison, more than half of the POSHA-S database was married (55%), and almost half of these were parents (50%). Regarding working status, 28% of the Saudi respondents were working at the time the study was conducted, more than half were students (52%), and 1.5% were retired. In the POSHA-S database, 14% of the respondents were students, while 63% were workers. In terms of health and abilities, in the Saudi sample, physical health was rated in the lowest quartile (0–25^th^ percentile), with a mean of 32. However, mental health, ability to learn new things, and ability to speak were rated higher than those in the POSHA-S database; mental health and ability to learn were placed in the fourth quartile (75^th^–100^th^ percentile).

Fig 1 shows more positive than average OSS for *beliefs* but less positive than average OSS for *self-reactions* in the Saudi sample. Thus, the OSS for the Saudi sample was almost the same as the POSHA-S database average.

Table 2 presents the subscores, components, items, OSS, and the generated percentiles for the Saudi sample. The OSS for the Saudi sample was 18 (36^th^ percentile), similar to the median of 19 for the POSHA-S database. By quartile, almost two-thirds (63%) of the Saudi respondents’ ratings were in the interquartile range, 23% in the first quartile, and 13% in the fourth quartile. Therefore, the average scores of the Saudi respondents were expected to fall within the 25^th^–75^th^ percentile.

**Table 2:** Self-identification and impression towards (Intelligence-Obesity-Mental illness–Left-handed–Stuttering: *POSHA-S mean values of the Saudi respondents*, *mean values from the POSHA-S database samples (268 samples circa March 2021)*, and Percentiles for the Saudi means relative to the POSHA-S database.

Variables	Saudi Respondents	Public POSHA-S samples (Median)	Public POSHA-S samples (Mean)	Saudi Sample Percentile
**Overall stuttering score (-100 to +100)**	18	19	17	36
**Beliefs about PWS (-100 to +100)**	22	33	33	23
**Traits and Personality**	**4**	**22**	**18**	**23**
Have themselves to blame*	88	84	70	57
Nervous or excitable*	-15	-2	-1	28
Shy or fearful*	-62	-17	-16	7
**PWS can be helped by**	**7**	**16**	**17**	**22**
*Other people who stutter*	-20	-3	-3	24
*SLP*	89	95	90	29
Doctor*	-49	-38	-36	31
**Causes of stuttering**	**13**	**34**	**31**	**11**
*Genetic inheritance*	20	17	17	44
Learning and Habits*	19	28	17	45
Emotional/Trauma*	-52	-1	-5	16
Act of God*	-69	64	39	4
Virus/disease*	68	39	35	77
Ghosts/demon*	89	90	83	44
**Potentials**	**66**	**66**	**64**	**44**
*Can make friends*	87	93	88	27
*Can lead normal lives*	87	90	85	36
*Can do any job*	86	53	46	93
*Should have jobs requiring judgment*	5	42	40	8
**Self-Reactions to PWS(-100 to +100)**	**13**	**3**	**1**	**70**
**Accommodating and Helping**	**53**	**44**	**40**	**59**
*Act like the person was talking normally*	95	84	78	84
Make a joke about stuttering*	89	91	84	39
Fill in the person’s words*	57	35	25	65
Should try to hide their stuttering*	82	76	68	55
Tell the person to “slow down” or “relax”*	23	14	9	51
*I Should help*	-26	-32	-29	40
**Social Distance and Sympathy**	**33**	**19**	**12**	**80**
*Feel comfortable or relaxed*	86	36	30	97
Feel pity*	35	16	14	71
Feel impatient*	91	62	55	92
Concern about doctor stuttering*	47	47	36	49
Concern about neighbor stuttering*	80	77	61	47
Concern about brother ‏or sister stuttering*	17	5	3	59
Concern about me stuttering*	-22	-33	-29	62
*Want to be stuttering*	-75	-66	-65	22
*Impression about stuttering*	38	2	1	97
**Knowledge and Experience**	**-37**	**-34**	**-33**	**31**
*Known amount about stuttering*	-22	-31	-32	48
*Known PWS*	-87	-86	-84	35
*Personal experience (me*, *family*, *friends)*	0	18	15	32
**Knowledge Source**	**4**	**-11**	**-12**	**64**
*Television*, *radio*, *films*	21	12	8	62
*Print*	-30	-14	-11	28
*Internet*	58	-27	-25	93
*School*	6	-9	-9	47
*Doctor*, *nurse specialist*	-35	-31	-26	41

The first part of Table 2 provides subscores of beliefs about the items and components related to PWS. Regarding beliefs about stuttering, the ratings were lower than the median of the POSHA-S database for all items except “have themselves to blame” and “genetic inheritance or virus/disease,” as causes of stuttering, and “can do any job.” In particular, the items rated inaccurately or negatively included nervous or excitable (−15; 28^th^ percentile), shy or fearful (−62; 7^th^ percentile), other PWS (−20; 24^th^ percentile), and doctors (−49; 31^st^ percentile). Regarding beliefs about the cause of stuttering, the following items were rated inaccurately or negatively: emotional trauma (−52; 16^th^ percentile) and act of God (−69; 4^th^ percentile). Fig 1 further shows the means of individual items within each component in relation to the median of the POSHA-S database. The participants responded more accurately to “not blaming PWS for their stuttering” (88; 57^th^ percentile). They also gave accurate responses for the following causes of stuttering: genetic inheritance (20; 44^th^ percentile), learning habits (19; 45^th^ percentile), virus or disease (68; 77^th^ percentile), and ghosts or demons (89; 44^th^ percentile). In other words, the respondents accepted that stuttering is caused by genetic inheritance, emotional trauma, or an act of God and rejected learning or habits, virus or disease, and ghosts or demons as possible causes. The beliefs of the respondents about the potential of PWS were accurate, including “can make friends” (87; 27^th^ percentile), “leading normal lives” (87; 36^th^ percentile), “doing any job” (86; 93^rd^ percentile), and “having jobs requiring judgment” (42; 8^th^ percentile).

The second part of Table 2 provides subscores of the self-reaction items and their components. The respondents rated most of the items positively, except for the following: providing help (−26; 40^th^ percentile), concern about me stuttering (−22; 62^nd^ percentile), wanting to stutter (−75; 22^nd^ percentile), knowing something about stuttering (−22; 48^th^ percentile), and knowing PWS (−87; 35^th^ percentile). Furthermore, magazines, newspapers, and books (−30; 28^th^ percentile) and doctors, nurses, and specialists (-35; 41^st^ percentile) were also rated negatively. With a high mean rating, the Saudi respondents held more positive than average self-reactions regarding the item “try to act like the person was talking normally” (95; 84^th^ percentile). Within the interquartile range, the Saudi respondents were more likely to reject “make a joke about stuttering” (89; 39^th^ percentile) and to ask PWS to “hide their stuttering” (82; 55^th^ percentile). They were also likely to reject, but less likely, to “fill in the person’s words” (57; 65^th^ percentile), to tell the person to “slow down or relax” (23; 51^st^percentile), and to offer help (−26; 40^th^ percentile).

In terms of social distance and sympathy, positive attitudes were observed regarding most items except “concern about me stuttering” (−22; 62^nd^ percentile) and “want to stutter” (−75; 22^nd^ percentile). Most of the respondents’ ratings were above the database median and included the following items: “feel comfortable and relaxed” (86; 97^th^ percentile), “feel pity” (35; 71^st^ percentile), “feel impatient” (91; 92^nd^ percentile), “concern about neighbor stuttering” (80; 47^th^ percentile), and “impression about stuttering” (38; 97^th^ percentile).

Negative ratings were observed for a known amount of stuttering (−22; 48^th^ percentile) and known PWS (−87; 35^th^ percentile). Regarding knowledge sources, the following items were the only ones rated positively: television, radio, or films (21; 62^nd^ percentile); Internet (58; 93^rd^ percentile); and schools (6; 47^th^ percentile).

### Predictors of the Overall Stuttering Score (OSS)

As shown in Table 3, the first two models were not significantly related to the OSS, and each model explained only 8% of the outcome variance [(R^2^ = .084, p = .12 (&) R^2^ = .085, p = .18)]. However, working status in both models was a significant OSS predictor (ß = −0.26, t = −2.1, p = .04). In the third model, both working status and multilingualism were significant OSS predictors [(ß = .29, t = 2.34, p = .02) & (ß = .18, t = 1.95, p = .05)], adding to the amount of explained variance (△R2 = 0.031, F = 1.8, p = 0.08).

**Table 3:** Hierarchical regression analysis to factors predicting OSS.

MODEL	Predictors	Coefficients ^a^						R^2^	R^2 change^	F	P
		B	ß	T	Sig.	95.0% CI for B					
						Lower	Upper				
**Model 1**	**(Constant)** ^**b**^	24.44		2.13	.04	1.65	47.22	.084	.084	1.721	.12
	Age (y)	-.010	-.01	-.03	.97	-.616	.596				
	Gender	-.178	-.02	-.04	.97	-8.485	8.129				
	Education(y)	.688	.09	.93	.36	-.780	2.157				
	Parental Status	-3.21	-.08	-.58	.57	-14.28	7.848				
	Working Status	-5.36	-.26	-2.1	.04*	-10.37	-.342				
	Income	.001	.001	.01	.99	-.112	.114				
**Model 2**	**(Constant)** ^**c**^	25.42		2.13	.04	1.816	49.021	.085	.001	1.480	.18
	Age (y)	-.02	-.01	-.06	.95	-.630	.592				
	Gender	-.27	-.01	-.07	.94	-8.633	8.087				
	Education(y)	.71	.09	.95	.34	-.769	2.193				
	Parental Status	-3.32	-.08	-.59	.56	-14.442	7.806				
	Working Status	-5.34	-.26	-2.10	.04*	-10.374	-.300				
	Income	.002	.004	.04	.96	-.112	.117				
	Health/Abilities	-.016	-.03	-.34	.74	-.109	.077				
**Model 3**	**(Constant)** ^**d**^	-2.430		-.13	.89	-39.106	34.245	.116	.031	1.803	.08
	Age (y)	.083	.04	.27	.79	-.529	.695				
	Gender	-1.459	-.03	-.35	.73	-9.803	6.886				
	Education(y)	.767	.11	1.04	.30	-.696	2.231				
	Parental Status	-3.908	-.09	-.70	.48	-14.911	7.096				
	Working Status	-5.927	-.29	-2.34	.02*	-10.938	-.916				
	Income	.00	.001	.01	.99	-.112	.113				
	Health/Abilities	-.018	-.04	-.38	.70	-.110	.074				
	Multilingual	14.19	.18	1.95	.05*	-.236	28.62				
**Model 4**	**(Constant)** ^**e**^	-5.5		-.29	.77	-42.31	31.39	.130	.014	1.803	.05*
	Age (y)	.121	.06	.39	.69	-.492	.734				
	Gender	-1.9	-.04	-.45	.66	-10.225	6.461				
	Education(y)	.80	.11	1.09	.28	-.657	2.263				
	Parental Status	-4.1	-.09	-.73	.47	-14.99	6.946				
	Working Status	-6.2	-.30	-2.4	.02*	-11.18	-1.163				
	Income	-.001	-.002	-.03	.98	-.114	.111				
	Health/Abilities	-.013	-.03	-.29	.77	-.105	.079				
	Multilingual	13.5	.17	1.86	.07	-.879	27.95				
	Self-identification	2.07	.12	1.31	.19	-1.064	5.197				
**Model 5**	**(Constant)** ^**f**^	21.2		1.05	.29	-18.96	61.29	.192	.062	2.558	< .001*
	Age (y)	.240	.119	.79	.43	-.359	.839				
	Gender	-3.29	-.08	-.80	.43	-11.42	4.850				
	Education(y)	.474	.065	.66	.51	-.958	1.906				
	Parental Status	-4.2	-.10	-.78	.44	-14.78	6.467				
	Working Status	-6.4	-.31	-2.59	.01*	-11.203	-1.499				
	Income	-.007	-.01	-.13	.90	-.116	.102				
	Health/Abilities	.010	.02	.21	.83	-.081	.100				
	Multilingual	10.6	.134	1.49	.14	-3.501	24.71				
	Self-identification	1.91	.110	1.25	.22	-1.124	4.94				
	No Person Known	-4.302	-.23	-2.88	.01*	-7.266	-1.34				
**Model 6**	**(Constant)** ^**g**^	10.349		.77	.44	-16.31	37.05	.648	.457	17.920	< .001*
	Age (y)	-.068	-.03	-.338	.74	-.469	.332				
	Gender	-4.026	-.09	-1.48	.14	-9.420	1.369				
	Education(y)	-.081	-.01	-.169	.87	-1.035	.873				
	Parental Status	3.093	.076	.858	.39	-4.054	10.239				
	Working Status	-2.182	-.11	-1.314	.19	-5.474	1.110				
	Income	.004	.01	.103	.92	-.068	.076				
	Health and Abilities	.001	.002	.028	.98	-.059	.061				
	Multilingual	5.65	.071	1.19	.24	-3.734	15.07				
	Self-identification	1.37	.079	1.4	.18	-.646	3.379				
	No Person Known	-2.69	-.16	-2.69	.01*	-4.673	-.706				
	Beliefs about PWS	.59	.724	11.8	.000*	.492	.691				
**Model 7**	**(Constant)** ^**h**^	.06		.139	.89	-.818	.941	1.00	.351	2.341	< .001*
	Age (y)	.007	.004	1.11	.27	-.006	.021				
	Gender	-.14	-.003	-1.56	.12	-.322	.037				
	Education(y)	.002	.000	.134	.89	-.029	.034				
	Parental Status	-.07	-.002	-.569	.57	-.304	.168				
	Working Status	-.03	-.002	-.59	.55	-.142	.076				
	Income	.003	.004	2.31	.02*	.000	.005				
	Health/Abilities	1.73	.000	.017	.98	-.002	.002				
	Multilingual	.092	.001	.59	.56	-.219	.402				
	Self-identification	.021	.001	.63	.52	-.045	.088				
	No Person Known	-.007	.00	-.20	.84	-.074	.061				
	Beliefs PWS	.49	.60	292.1	< .001*	.489	.496				
	Self-Reactions to PWS	.49	.66	314.3	< .001*	.496	.502				

* P ≤ 0.05 is significance; B: unstandardized beta" regression coefficient"; β: standardized beta, a: Dependent variable: OSS: Overall Stuttering score

b. Predictors: (Constant), Age (y), Gender Education(y), Marital Status, Parental Status, Working Status, Income, c. Predictors: (Constant), Age (y), Gender Education(y), Marital Status, Parental Status, Working Status, Income, health/ abilities, d. Predictors: (Constant), Age (y), Gender Education(y), Marital Status, Parental Status, Working Status, Income, health & abilities, Multilingual, e. Predictors: (Constant), Age (y), Gender Education(y), Marital Status, Parental Status, Working Status, Income, health & abilities, Multilingual, Self-identification, f. Predictors: (Constant), Age (y), Gender Education(y), Marital Status, Parental Status, Working Status, Income, health & abilities, Multilingual, Self-identification, No Person Known, g. Predictors: (Constant), Age (y), Gender Education(y), Marital Status, Parental Status, Working Status, Income, health & abilities, Multilingual, Self-identification, No Person Known, Beliefs about PWS, h. Predictors: (Constant), Age (y), Gender Education(y), Marital Status, Parental Status, Working Status, Income, health & abilities, Multilingual, Self-identification, No Person Known, Beliefs about PWS, Self-Reactions to PWS, i. Predictors: (Constant), Age (y), Gender Education(y), Marital Status, Parental Status, Working Status, Income, health & abilities, Multilingual, Self-identification, No Person Known, Beliefs about PWS, Self-Reactions to PWS, Obesity/Mental illness

The fourth model significantly predicted 13% of the variance in the OSS (R^2^ = .13, p = .05), with working status as a significant predictive factor (ß = −.3, t = −2.4, p = .02), significantly adding to the amount of explained variance (△R2 = 0.014, F = 1.80, p = .05). In the fifth model, both working status and no person known items were significant predictive factors of the OSS [(ß = .31, t = 2.59, p = .01) & (ß = .23, t = 2.88, p = .01)], adding to the amount of explained variance (△R2 = 0.062, F = 2.55, p < 0.001). Similarly, the sixth model significantly predicted 65% of the variance in the OSS (R^2^ = .648, p < .001), and both no person known and beliefs about PWS significantly predicted the OSS [(ß = 0.16, t = 2.69, p = .01) & (ß = 0.72, t = 11.78, p < .001)]. The seventh model revealed that income (ß = 0.004, t = 2.31, p = .02), beliefs about PWS (ß = 0.60, t = 292.9, p < .001), and self-reactions to PWS scores (ß = 0.66, t = 314.3, p < .001) were the substantial predictors of the OSS, significantly predicting 100.0% of the outcome variance (R^2^ = 1.0, p < .001).

## Discussion

In the current study, attitudes toward stuttering and PWS were investigated in a sample of the general population of Saudi adults, and their attitudes were compared with those of the reference POSHA-S database, which represents 268 samples from 47 countries. Further investigation was conducted to assess the associations among sociodemographic factors (such as age, gender, years of education, income, health abilities, multilingualism, and marital, parental, and working status) and the OSS.

Compared to the sampled population worldwide, the Saudi population held stuttering attitudes that were within the average range. In terms of the variables that could predict a positive versus negative OSS, the respondents’ working status and multilingualism were significant predictors. In contrast, the respondent’s age, gender, education, parental status, health, abilities, and income were not significant predictors of a more positive or more negative OSS.

### Attitudes toward stuttering

The OSS for the Saudi participants on the −100 to +100 scale was 18 units, which is similar to the score for the POSHA-S database (19) and for a Polish sample [7, 27]. However, comparing Saudi and Egyptian scores revealed that Saudi people had a more positive attitude toward stuttering and PWS by 14 units (OSS = 4) [3]. Compared to the POSHA-S database, 63.33% of the Saudi respondents’ ratings were in the interquartile range, 23% in the first quartile, and 13% in the fourth quartile (circa March 2021).

The Saudi respondents believed that PWS are shy, fearful, nervous, or excitable. Such beliefs are expected as these characteristics are considered symptoms of “stuttering stereotypes” [19] and have been widely reported [4, 7, 16]. Regarding beliefs about stuttering, the Saudi respondents accurately identified who should help PWS, with 89% of the respondents believing that a speech-language pathologist (SLP) should help PWS, which indicates an evolving appreciation of and confidence in the SLP profession internationally, including in the KSA. The Saudi respondents were also less likely to advocate for help from a medical doctor. The high ratings for the belief that SLPs or doctors can help PWS align with the ratings in the POSHA-S database. Furthermore, the Saudi population was more likely to believe PWS should help each other than other populations in the POSHA-S sample.

Public awareness of stuttering is essential as public knowledge and beliefs about its origin can affect whether help and treatment for the problem are sought and from whom. In the current study, most Saudi respondents believed that stuttering is an act of God, which accords with the findings reported for Kuwaiti, Egyptian, and Turkish samples [3, 4, 22, 29]. The study also revealed that Saudis believe that stuttering is a result of emotional trauma, as reported in other studies [22, 30], indicating confusion around the causes of the disorder. Also, in line with the findings reported by other studies[22, 31], less than half of the Saudi sample related the cause of stuttering to genetics. In contrast, most of the sample accurately rejected that stuttering is caused by learning and habits, viruses and diseases, ghosts and demons, which aligns with the other samples in the POSHA-S database.

Most Saudi respondents had positive beliefs about the potential of PWS to make friends, lead normal lives, and have jobs requiring judgment. Regarding job opportunities for PWS, the respondents’ ratings were higher than those in the POSHA-S sample; thus, the Saudi respondents did not believe that stuttering should interfere with job opportunities or a normal social life. These positive beliefs could reflect the efforts of the Saudi vision for the community to develop, the mentioned above, civic values. Regarding self-reactions to PWS, slight differences were noted when comparing the Saudi sample with the POSHA-S database. Almost all respondents (95%) stated that they would behave as if the person speaks normally (fourth quartile). Similar to the median scores for the POSHA-S sample, the Saudi respondents rejected making jokes about stuttering, filling in the person’s words, asking them to hide their stuttering, telling them to slow down or relax, and not helping PWS (all in the third quartile).

Regarding social distance and sympathy, the Saudi respondents had a considerably more positive impression of stuttering than the median of the POSHA-S database—a finding also supported by other studies [4, 32]. The respondents rejected feeling pity toward someone who stuttered or was impatient and were not concerned about a doctor, neighbor, or sibling stuttering. Such positive attitudes were also noted in the POSHA-S database but less prominently. Despite such positive attitudes toward stuttering, the Saudi population’s rate of wanting to stutter was more negative than that of the POSHA-S sample.

Concerning knowledge sources about stuttering, the primary source was the Internet (58%), followed by television, radio, and film (21%), and, finally, school (6%). In contrast, knowledge obtained from scholarly publications and specialists was limited (−30% and −35%, respectively). In contrast, for the POSHA-S database, the primary sources were television, radio, and films (12%). The high rates assigned to the Internet as the primary source of knowledge in the current study are noteworthy since many studies have reported that Internet sources impact the health knowledge of the Saudi population [33, 34]. This finding indicates that social media likely plays a part in how Saudi individuals obtain information and knowledge about different disorders.

Regarding knowledge and experience of stuttering, the respondents had ratings similar to those for the POSHA-S database (31^st^ percentile). Initially, it was assumed that the positive attitudes among the Saudi respondents were related to their level of education; hence, increased education was associated with more positive attitudes toward PWS [28] However, this assumption was discarded as the findings revealed that education did not predict the OSS. It is possible that the positive attitudes were related to the respondents’ working or studying status, as people who do not stutter may encounter PWS at their workplaces or schools. Despite the Saudi population’s relatively more positive attitude toward stuttering than the POSHA-S sample, the amount of stuttering knowledge rating remained low for both the Saudi and POSHA-S samples. This is surprising given that the respondents gave accurate responses regarding who should help PWS (i.e., SLPs), though they also thought (inaccurately) that a medical doctor could help PWS. Therefore, a sufficient amount of correct stuttering knowledge needs to be presented on the Internet and social media. More education and awareness surrounding stuttering and PWS conveyed through educational websites, films, and television programs may yield better knowledge on the topic, positively impacting from whom and where one seeks help for the problem. Students, classroom educators in early grade levels, and medical personnel in health centers for various ages may particularly benefit from additional education about and exposure to stuttering.

### Sociodemographic predictors of OSS

The current study demonstrated that working or studying status and multilingualism were significant predictors of OSS, which is in line with Valente et al. [28], who found that respondents who spoke more than one language had a more positive attitude toward PWS. The strong positive relationship between the OSS and employment or speaking more than one language may be attributed to people who do not stutter dealing with PWS at their workplace or school. In contrast, the respondents’ age, gender, education, parental status, health, abilities, and income were not significant predictors of better or worse OSS. The lack of a relationship between age and the OSS in the current study is in line with that conducted on a Turkish sample [29]. However, it does not support a study conducted on a Portuguese sample [28], which revealed that the older age group had less positive attitudes than the younger age group.

Previous research has reported controversial findings regarding respondent gender as an OSS predictor. While some studies have reported gender as a significant predictor of attitudes toward stuttering [35, 36], the very small effect sizes in these studies made their findings difficult to interpret. Other studies, including the current one, have found that the respondents’ gender did not predict the OSS [28]. For the Saudi sample, education also did not predict the OSS, which is in contrast to the findings of Valente et al. [28], who demonstrated that more positive attitudes are associated with high levels of education. Supporting the claim that parental status and attitudes are unrelated, St.Louis et al. [37]and Valente et al. [28] showed that being a parent or nonparent resulted in very similar attitudes regarding the appraisal of the Stuttering Environment (ASE), a tool similar to POSHA-S. The results further revealed that income is not a predictor of better or worse OSS. This finding contrasts with that of Arafa [3], who observed that people with an average to high income have a better OSS than those with a low income.

Despite its significant contributions to understanding the attitudes of the general population in the KSA toward PWS, the study also had some notable limitations. Although 99% of Saudi Arabians have Internet access and more than 79% use social media [38], reliance on social media to recruit participants for the current study may be a limitation as it excludes people who are not computer-linked from the sample. Future studies should use other survey methods combined with social media to obtain more descriptive results. Furthermore, the current sample is predominantly female, which may limit the generalizability of the findings. As reports have shown that online surveys are more likely to be self-selected by females [39, 40], future studies could attempt to resolve the gender imbalance by, for example, distributing the survey at men’s sports centers or in paper forms at senior centers. A campaign could include press releases from specialist SLPs to promote accurate information about stuttering. Finally, although the current study supports previous work demonstrating that gender, parental status, and marital status do not predict better or worse OSS, additional intervening variables that predict the OSS may exist and should be investigated in the future.

## Conclusion

The findings obtained regarding attitudes toward and reactions to stuttering in the Saudi population are similar to those in the POSHA-S database, with Saudi respondents expressing similar impressions, beliefs, and self-reactions toward PWS to the median of the database. This suggests that Saudi people generally hold sensitive attitudes toward PWS. However, the population’s knowledge about stuttering tends to be limited [22, 41], making it important to disseminate stuttering information via knowledge sources widely used in the Saudi Arabian community, such as the Internet and social media. Including press releases from specialist SLPs in campaigns may also effectively promote accurate information about stuttering and the services provided by the SLPs for PWS. A greater level of education and awareness relating to stuttering and PWS could also be achieved through the use of educational websites, films, and television programs, which might positively impact whom and where one seeks assistance for the disorder.

## Supporting information

S1 Data (XLSX)
